# Supplementary material for: Plasmid Curing and Exchange Using a Novel Counter-Selectable Marker Based on Unnatural Amino Acid Incorporation at a Sense Codon
Source: Int J Mol Sci. 2021 Oct 25;22(21):11482. doi: 10.3390/ijms222111482 (PMC8583848; doi:10.3390/ijms222111482)
Supplement: Supplementary file 1 [file ijms-22-11482-s001.zip › ijms-1417242-supplementary.pdf]

Supporting Information

Plasmid curing and exchange using a novel counter-selectable marker based on unnatural amino acid incorporation at a sense codon

Yusuke Kato

Institute of Agrobiological Sciences, National Agriculture and Food Research Organization (NARO), Oowashi 1 - 2, Tsukuba, Ibaraki 305 - 8634, Japan; kato@affrc.go.jp

Figure S1.

Nucleotide sequence of the target plasmid used in the plasmid curing experiment shown in Figure 1.

1 ttggtagctc agagaacctt cgaaaaaccg ccctgcaagg cggtttttc gttttcagag
61 caagagatta cgcgcagacc aaaacgatct caagaagatc atcttattaa tcagataaaa
121 tatttctaga tttcagtgca atttatctct tcaaatgtag cacctgaagt cagccccata
181 cgatataagt tgtaattctc atgtttgaca gcattatcat cgataagctt taatgcggta
241 gtttatcaca gttaaattgc taacgcagtc aggcaccgtg tatgaaatct aacaatgcgc
301 tcatcgtcat cctcggcacc gtcaccctgg atgctgtagg cataggcttg gttatgccgg
361 tactgccggg cctcttgcgg gatatcgtcc attccgacag catcgccagt cactatggcg
421 tgctgctagc gctatatgcg ttgatgcaat ttctatgcgc acccgttctc ggagcactgt
481 ccgaccgctt tggccgccgc ccagtcctgc tcgcttcgct acttggagcc actatcgact
541 acgcgatcat ggcgaccaca cccgtcctgt gcatcctcta cgccggacgc atcgtggccg
601 gcatcaccgg cgccacaggt gcggttgctg gcgcctatat cgccgacatc accgatgggg
661 aagatcgggc tcgccacttc gggctcatga gcgcttgttt cggcgtgggt atggtggcag
721 gccccgtggc cgggggactg ttgggcgcca tctccttgca tgcaccattc cttgcggcgg
781 cggtgctcaa cggcctcaac ctactactgg gctgcttcct aatgcaggag tcgcataagg
841 gagagcgtcg aagctttaat gcggtagttt atcacagtta aattgctaac gcagtcaggc
901 accgtgtatg aaatctaaca atgcgctcat cgtcatcctc ggcaccgtca ccctggatgc
961 tgtaggcata ggcttggtta tgccggtact gccgggcctc ttgcgggata tcgtccattc
1021 cgacagcatc gccagtcact atggcgtgct gctagcatcg acgagtctgg ctttgacatt
1081 cgactagaag tggacggtgg cgtgaaggtg aacaacattg gcgaaatcgc tgcggcgggc
1141 gcggatatgt tcgtcgccgg ttcggcaatc ttcgaccagc cagactacaa aaaagtcatt
1201 gatgaaatgc gcagtgaact ggcaaaggta agtcatgaat aagtttgaag atattcgcgg
1261 cgtcgctttt gatcttgatg gtacgctggt cgacagtgct cctggtcttg ctgctgcggt
1321 agatatggcg ctgtatgcgc tggagttgcc cgtcgcaggt gaagaacgcg ttattacctg
1381 gattggtaac ggcgcagatg ttctgatgga gcgcgcattg acctgggcgc gtcaggaacg
1441 tgcgactcag cgtaaaacaa tgggtaaacc gcccgttgat gacgacattc cggcagaaga
1501 acaggtacgt attctgcgta aactgttcga tcgctactat ggcgaggttg ccgaagaggg
1561 gacgtttttg ttcccgcacg ttgccgatac gttgggcgcg ttgcaggcta aaggcctgcc
1621 gctaggcctg gtcaccaaca aaccgacgcc gttcgtcgcg ccgctgctcg aagccttaga
1681 tatcgccaaa tacttcagcg cggtgattgg tggtgatgat gtgcaaaaca aaaaaccgca
1741 tccggacccg ctgttactgg tggctgagcg gatgggaatt gccccacaac agatgctgtt
1801 tgtcggcgac tcacgcaatg atattcaggc ggcaaaagcg gcaggttgcc catcagttgg
1861 cttaacctac ggatataact acggcgaggc tatcgatctc agccagcctg atgtaattta
1921 tcagtctata aatgaccttc tgcccgcatt agggcttccg catagcgaaa atcaggaatc
1981 gacatATGGA TAAAAAACCA CTAAACACTC TGATATCTGC AACCGGGCTC TGGATGTCCA
2041 GGACCGGAAC AATTCATAAA ATAAAACACC ACGAAGTCTC TCGAAGCAAA ATCTATATTG

2101 AAATGGCATG CGGAGACCAC CTTGTTGTAA ACAACTCCAG GAGCAGCAGG ACTGCAAGAG
2161 CGCTCAAGCA CCACAAATAC AGGAAGACCT GCAAACGCTG CAGGGTTTCG GATGAGGATC
2221 TCAATAAGTT CCTCACAAAG GCAAACGAAG ACCAGACAAG CGTAAAAGTC AAGGTCGTTT
2281 CTGCCCCTAC CAGAACGAAA AAGGCAATGC CAAAATCCGT TGCGAGAGCC CCGAAACCTC
2341 TTGAGAATAC AGAAGCGGCA CAGGCTCAAC CTTCTGGATC TAAATTTTCA CCTGCGATAC
2401 CGGTTTCCAC CCAAGAGTCA GTTTCTGTCC CGGCATCTGT TTCAACATCA ATATCAAGCA
2461 TTTCTACAGG AGCAACTGCA TCCGCACTGG TAAAAGGGAA TACGAACCCC ATTACATCCA
2521 TGTCTGCCCC TGTTCAGGCA AGTGCCCCCG CACTTACGAA GAGCCAGACT GACAGGCTTG
2581 AAGTCCTGTT AAACCCAAAA GATGAGATTT CCCTGAATTC CGGCAAGCCT TTCAGGGAGC
2641 TTGAGTCCGA ATTGCTCTCT CGCAGAAAAA AAGACCTGCA GCAGATCTAC GCGGAAGAAA
2701 GGGAGAATTA TCTGGGGAAA CTCGAGCGTG AAATTACCAG GTTCTTTGTG GACAGGGGTT
2761 TTCTGGAAAT AAAATCCCCG ATCCTGATCC CTCTTGAGTA TATCGAAAGG ATGGGCATTG
2821 ATAATGATAC CGAACTTTCA AAACAGATCT TCAGGGTTGA CAAGAACTTC TGCCTGAGAC
2881 CCATGCTTGC TCCAAACCTT GCCAACTACC TGCGCAAGCT TGACAGGGCC CTGCCTGATC
2941 CAATAAAAAT TTTTGAAATA GGCCCATGCT ACAGAAAAGA GTCCGACGGC AAAGAACACC
3001 TCGAAGAGTT TACCATGCTG AACTTCTGCC AGATGGGATC GGGATGCACA CGGGAAAATC
3061 TTGAAAGCAT AATTACAGAC TTCCTGAACC ACCTGGGAAT TGATTTCAAG ATCGTAGGCG
3121 ATTCCTGCAT GGTCTTTGGG GATACCCTTG ATGTAATGCA CGGAGACCTG GAACTTTCCT
3181 CTGCAGTAGT CGGACCCATA CCGCTTGACC GGGAATGGGG TATTGATAAA CCCTGGATAG
3241 GGGCAGGTTT CGGGCTCGAA CGCCTTCTCA AGGTTAAACA CGACTTTAAA AATATCAAGA
3301 GAGCTGCAAG GTCCGGGTCT TACTATAACG GGATTTCTAC CAACCTGTAA ggatctgcat
3361 cgcaggatgc tgctggctac cctgtggaac acctacatct gtattaacga agcgctggca
3421 ttgaccctga gtgatttttc tctggtcccg ccgcatccat accgccagtt gtttaccctc
3481 acaacgttcc agtaaccggg catgttcatc atcagtaacc cgtatcgtga gcatcctctc
3541 tcgtttcatc ggtatcatta cccccatgaa cagaaatccc ccttacacgg aggcatcagt
3601 gaccaaacag gaaaaaaccg cccttaacat ggcccgcttt atcagaagcc agacattaac
3661 gcttctggag aaactcaacg agctggacgc ggatgaacag gcagacatct gtgaatcgct
3721 tcacgaccac gcatcaaaaa aaatccttag ctttcgctaa ggatctgcag TGGCGGAAAC
3781 CCCGGGAATC TAACCCGGCT GAACGGATTG GCAGTCCATT CGATCTACAT GATCAGGTTT
3841 CCGGATCCGT TACAAGTATT ACACAAAGTT TTTTATGTTG AGAATATTTT TTTGATgggg
3901 cgccacttat ttttgatcgt tcgctcaaag aagcggcgcc gtcgaccgat gcccttgaga
3961 gccttcaacc cagtcagctc cttccggtgg gcgcggggca tgactatcgt cgccgcactt
4021 atgactgtct tctttatcat gcaactcgta ggacaggtgc cggcagcgct ctgggtcatt
4081 ttcggcgagg accgctttcg ctggagcgcg acgatgatcg gcctgtcgct tgcggtattc
4141 ggaatcttgc acgccctcgc tcaagccttc gtcactggtc ccgccaccaa acgtttcggc
4201 gagaagcagg ccattatcgc cggcatggcg gccgaggtct gcctcgtgaa gaaggtgttg

4261 ctgactcata ccaggcctga atcgccccat catccagcca gaaagtgagg gagccacggt
4321 tgatgagagc tttgttgtag gtggaccagt tggtgatttt gaacttttgc tttgccacgg
4381 aacggtctgc gttgtcggga agatgcgtga tctgatcctt caactcagTT ACGCCCCGCC
4441 CTGCCACTCA TCGCAGTACT GTTGTAATTC ATTAAGCATT CTGCCGACAT GGAAGCCATC
4501 ACAAACGGCA TGATGAACCT GAATCGCCAG CGGCATCAGC ACCTTGTCGC CTTGCGTATA
4561 ATATTTGCCC ATGGTGAAAA CGGGGGCGAA GAAGTTGTCC ATATTGGCCA CGTTTAAATC
4621 AAAACTGGTG AAACTCACCC AGGGATTGGC TGAGACGAAA AACATATTCT CAATAAACCC
4681 TTTAGGGAAA TAGGCCAGGT TTTCACCGTA ACACGCCACA TCTTGCGAAT ATATGTGTAG
4741 AAACTGCCGG AAATCGTCGT GGTATTCACT CCAGAGCGAT GAAAACGTTT CAGTTTGCTC
4801 ATGGAAAACG GTGTAACAAG GGTGAACACT ATCCCATATC ACCAGCTCAC CGTCTTTCAT
4861 TGCCATACGG AATTCCGGAT GAGCATTCAT CAGGCGGGCA AGAATGTGAA TAAAGGCCGG
4921 ATAAAACTTG TGCTTATTTT TCTTTACGGT CTTTAAAAAG GCCGTAATAT CCAGCTGAAC
4981 GGTCTGGTTA TAGGTACATT GAGCAACTGA CTGAAATGCC TCAAAATGTT CTTTACGATG
5041 CCATTGGGAT ATATCAACGG TGGTATATCC AGTGATTTTT TTCTCCATtt tagcttcctt
5101 agctcctgaa aatctcgata actcaaaaaa tacgcccggt agtgatctta tttcattatg
5161 gtgaaagttg gaacctctta cgtgccgatc aacgtctcat tttcgccaaa agttggccca
5221 gggcttcccg gtatcaacag ggacaccagg atttatttat tctgcgaagt gatcttccgt
5281 cacaggtatt tattcggcgc aaagtgcgtc gggtgatgct gccaacttac tgatttagtg
5341 tatgatggtg tttttgaggt gctccagtgg cttctgtttc tatcagctgt ccctcctgtt
5401 cagctactga cggggtggtg cgtaacggca aaagcaccgc cggacatcag cgctagcgga
5461 gtgtatactg gcttactatg ttggcactga tgagggtgtc agtgaagtgc ttcatgtggc
5521 aggagaaaaa aggctgcacc ggtgcgtcag cagaatatgt gatacaggat atattccgct
5581 tcctcgctca ctgactcgct acgctcggtc gttcgactgc ggcgagcgga aatggcttac
5641 gaacggggcg gagatttcct ggaagatgcc aggaagatac ttaacaggga agtgagaggg
5701 ccgcggcaaa gccgtttttc cataggctcc gcccccctga caagcatcac gaaatctgac
5761 gctcaaatca gtggtggcga aacccgacag gactataaag ataccaggcg tttcccctg
5821 gcggctccct cgtgcgctct cctgttcctg cctttcggtt taccggtgtc attccgctgt
5881 tatggccgcg tttgtctcat tccacgcctg acactcagtt ccgggtaggc agttcgctcc
5941 aagctggact gtatgcacga accccccgtt cagtccgacc gctgcgcctt atccggtaac
6001 tatcgtcttg agtccaaccc ggaaagacat gcaaaagcac cactggcagc agccactggt
6061 aattgattta gaggagttag tcttgaagtc atgcgccggt taaggctaaa ctgaaaggac
6121 aagttttggt gactgcgctc ctccaagcca gttacctcgg ttcaaagag

Comments

ZKRS ORF: 1986-3350 (highlighted in pink)

$tRNA^{Pyl}_{GCC}$: 3771-3896 (complementary strand; highlighted in deep green. The anticodon region is highlighted in yellow-green)

cat ORF: 4429-5088 (complementary strand; highlighted in light blue)

p15A origin: 5450-192 (highlighted in gray)

Figure S2.

Nucleotide sequence of the target plasmid used in the plasmid exchange experiment shown in Figure 2. The positions and sequences of the PCR primers specific for the target plasmids (ZKRS-s and ZKRS-as) used in Figure 2e are also shown.

1 ttggtagctc agagaacctt cgaaaaaccg ccctgcaagg cggttttttc gttttcagag
61 caagagatta cgcgcagacc aaaacgatct caagaagatc atcttattaa tcagataaaa
121 tatttctaga tttcagtgca atttatctct tcaaatgtag cacctgaagt cagccccata
181 cgatataagt tgtaattctc atgtttgaca gcattatcat cgataagctt taatgcggta
241 gtttatcaca gttaaattgc taacgcagtc aggcaccgtg tatgaaatct aacaatgcgc
301 tcatcgtcat cctcggcacc gtcaccctgg atgctgtagg cataggcttg gttatgccgg
361 tactgccggg cctcttgcgg gatatcgtcc attccgacag catcgccagt cactatggcg
421 tgctgctagc gctatatgcg ttgatgcaat ttctatgcgc acccgttctc ggagcactgt
481 ccgaccgctt tggccgccgc ccagtcctgc tcgcttcgct acttggagcc actatcgact
541 acgcgatcat ggcgaccaca cccgtcctgt gcatcctcta cgccggacgc atcgtggccg
601 gcatcaccgg cgccacaggt gcggttgctg gcgcctatat cgccgacatc accgatgggg
661 aagatcgggc tcgccacttc gggctcatga gcgcttgttt cggcgtgggt atggtggcag
721 gccccgtggc cgggggactg ttgggcgcca tctccttgca tgcaccattc cttgcggcgg
781 cggtgctcaa cggcctcaac ctactactgg gctgcttcct aatgcaggag tcgcataagg
841 gagagcgtcg aagctttaat gcggtagttt atcacagtta aattgctaac gcagtcaggc
901 accgtgtatg aaatctaaca atgcgctcat cgtcatcctc ggcaccgtca ccctggatgc
961 tgtaggcata ggcttggtta tgccggtact gccgggcctc ttgcgggata tcgtccattc
1021 cgacagcatc gccagtcact atggcgtgct gctagcatcg acgagtctgg ctttgacatt
1081 cgactagaag tggacggtgg cgtgaaggtg aacaacattg gcgaaatcgc tgcggcgggc
1141 gcggatatgt tcgtcgccgg ttcggcaatc ttcgaccagc cagactacaa aaaagtcatt
1201 gatgaaatgc gcagtgaact ggcaaaggta agtcatgaat aagtttgaag atattcgcgg
1261 cgtcgctttt gatcttgatg gtacgctggt cgacagtgct cctggtcttg ctgctgcggt
1321 agatatggcg ctgtatgcgc tggagttgcc cgtcgcaggt gaagaacgcg ttattacctg
1381 gattggtaac ggcgcagatg ttctgatgga gcgcgcattg acctgggcgc gtcaggaacg
1441 tgcgactcag cgtaaaacaa tgggtaaacc gcccgttgat gacgacattc cggcagaaga
1501 acaggtacgt attctgcgta aactgttcga tcgctactat ggcgaggttg ccgaagaggg
1561 gacgtttttg ttcccgcacg ttgccgatac gttgggcgcg ttgcaggcta aaggcctgcc
1621 gctaggcctg gtcaccaaca aaccgacgcc gttcgtcgcg ccgctgctcg aagccttaga
1681 tatcgccaaa tacttcagcg cggtgattgg tggtgatgat gtgcaaaaca aaaaaccgca
1741 tccggacccg ctgttactgg tggctgagcg gatgggaatt gccccacaac agatgctgtt
1801 tgtcggcgac tcacgcaatg atattcaggc ggcaaaagcg gcaggttgcc catcagttgg
1861 cttaacctac ggatataact acggcgaggc tatcgatctc agccagcctg atgtaattta
1921 tcagtctata aatgaccttc tgcccgcatt agggcttccg catagcgaaa atcaggaatc
1981 gacatATGGA TAAAAAACCA CTAAACACTC TGATATCTGC AACCGGGCTC TGGATGTCCA
2041 GGACCGGAAC AATTCATAAA ATAAAACACC ACGAAGTCTC TCGAAGCAAA ATCTATATTG

2101 AAATGGCATG CGGAGACCAC CTTGTTGTAA ACAACTCCAG GAGCAGCAGG ACTGCAAGAG
2161 CGCTCAAGCA CCACAAATAC AGGAAGACCT GCAAACGCTG CAGGGTTTCG GATGAGGATC
2221 TCAATAAGTT CCTCACAAAG GCAAACGAAG ACCAGACAAG CGTAAAAGTC AAGGTCGTTT
2281 CTGCCCCTAC CAGAACGAAA AAGGCAATGC CAAAATCCGT TGCGAGAGCC CCGAAACCTC
2341 TTGAGAATAC AGAAGCGGCA CAGGCTCAAC CTTCTGGATC TAAATTTTCA CCTGCGATAC
2401 CGGTTTCCAC CCAAGAGTCA GTTTCTGTCC CGGCATCTGT TTCAACATCA ATATCAAGCA
2461 TTTCTACAGG AGCAACTGCA TCCGCACTGG TAAAAGGGAA TACGAACCCC ATTACATCCA
2521 TGTCTGCCCC TGTTCAGGCA AGTGCCCCCG CACTTACGAA GAGCCAGACT GACAGGCTTG
2581 AAGTCCTGTT AAACCCAAAA GATGAGATTT CCCTGAATTC CGGCAAGCCT TTCAGGGAGC
2641 TTGAGTCCGA ATTGCTCTCT CGCAGAAAAA AAGACCTGCA GCAGATCTAC GCGGAAGAAA
2701 GGGAGAATTA TCTGGGGAAA CTCGAGCGTG AAATTACCAG GTTCTTTGTG GACAGGGGTT
2761 TTCTGGAAAT AAAATCCCCG ATCCTGATCC CTCTTGAGTA TATCGAAAGG ATGGGCATTG
2821 ATAATGATAC CGAACTTTCA AAACAGATCT TCAGGGTTGA CAAGAACTTC TGCCTGAGAC
2881 CCATGCTTGC TCCAAACCTT GCCAACTACC TGCGCAAGCT TGACAGGGCC CTGCCTGATC
2941 CAATAAAAAT TTTTGAAATA GGCCCATGCT ACAGAAAAGA GTCCGACGGC AAAGAACACC
3001 TCGAAGAGTT TACCATGCTG AACTTCTGCC AGATGGGATC GGGATGCACA CGGGAAAATC
3061 TTGAAAGCAT AATTACAGAC TTCCTGAACC ACCTGGGAAT TGATTTCAAG ATCGTAGGCG
3121 ATTCCTGCAT GGTCTTTGGG GATACCCTTG ATGTAATGCA CGGAGACCTG GAACTTTCCT
3181 CTGCAGTAGT CGGACCCATA CCGCTTGACC GGGAATGGGG TATTGATAAA CCCTGGATAG
3241 GGGCAGGTTT CGGGCTCGAA CGCCTTCTCA AGGTTAAACA CGACTTTAAA AATATCAAGA
3301 GAGCTGCAAG GTCCGGGTCT TACTATAACG GGATTTCTAC CAACCTGTAA ggatctgcat
3361 cgcaggatgc tgctggctac cctgtggaac acctacatct gtattaacga agcgctggca
3421 ttgaccctga gtgatttttc tctggtcccg ccgcatccat accgccagtt gtttaccctc
3481 acaacgttcc agtaaccggg catgttcatc atcagtaacc cgtatcgtga gcatcctctc
3541 tcgtttcatc ggtatcatta cccccatgaa cagaaatccc ccttacacgg aggcatcagt
3601 gaccaaacag gaaaaaaccg cccttaacat ggcccgcttt atcagaagcc agacattaac
3661 gcttctggag aaactcaacg agctggacgc ggatgaacag gcagacatct gtgaatcgct
3721 tcacgaccac gcatcaaaaa aatccttag ctttcgctaa ggatctgcag TGGCGGAAAC
3781 CCCGGGAATC TAACCCGGCT GAACGGATTG CGAGTCCATT CGATCTACAT GATCAGGTTT
3841 CCGGATCCGT TACAAGTATT ACACAAAGTT TTTTATGTTG AGAATATTTT TTTGATggac
3901 ccgtatcgtg agcatcctct ctcgtttcat cggtatcatt acccccatga acagaaatcc
3961 cccttacacg gaggcatcag tgaccaaaca ggaaaaaacc gcccttaaca tggcccgctt
4021 tatcagaagc cagacattaa cgcttctgga gaaactcaac gagctggacg cggatgaaca
4081 ggcagacatc tgtgaatcgc ttcacgacca cgcatcaaaa aaaatcctta gctttcgcta
4141 aggatctgca gTGGCGGAAA CCCCGGGAAT CTAACCCGGC TGAACGGATT ACCAGTCCAT
4201 TCGATCTACA TGATCAGGTT TCCGGATCCG TTACAAGTAT TACACAAAGT TTTTTATGTT

4261 GAGAATATTT TTTTGATGGG GCGCCACTTA TTTTTGATcg ttcgctcaaa gaagcggcgc
4321 cgtcgaccga tgcccttgag agccttcaac ccagtcagct ccttccggtg ggcgcggggc
4381 atgactatcg tcgccgcact tatgactgtc ttctttatca tgcaactcgt aggacaggtg
4441 ccggcagcgc tctgggtcat tttcggcgag gaccgctttc gctggagcgc gacgatgatc
4501 ggcctgtcgc ttgcggtatt cggaatcttg cacgccctcg ctcaagcctt cgtcactggt
4561 cccgccacca aacgtttcgg cgagaagcag gccattatcg ccggcatggc ggccgaggtc
4621 tgcctcgtga agaaggtgtt gctgactcat accaggcctg aatcgcccca tcatccagcc
4681 agaaagtgag ggagccacgg ttgatgagag ctttgttgta ggtggaccag ttggtgattt
4741 tgaacttttg ctttgccacg gaacggtctg cgttgtcggg aagatgcgtg atctgatcct
4801 tcaactcagT TACGCCCCGC CCTGCCACTC ATCGCAGTAC TGTTGTAATT CATTAAGCAT
4861 TCTGCCGACA TGGAAGCCAT CACAAACGGC ATGATGAACC TGAATCGCCA GCGGCATCAG
4921 CACCTTGTCG CCTTGCGTAT AATATTTGCC CATGGTGAAA ACGGGGGCGA AGAAGTTGTC
4981 CATATTGGCC ACGTTTAAAT CAAAACTGGT GAAACTCACC CAGGGATTGG CTGAGACGAA
5041 AAACATATTC TCAATAAACC CTTTAGGGAA ATAGGCCAGG TTTTCACCGT AACACGCCAC
5101 ATCTTGCGAA TATATGTGTA GAAACTGCCG GAAATCGTCG TGGTATTCAC TCCAGAGCGA
5161 TGAAAACGTT TCAGTTTGCT CATGGAAAAC GGTGTAACAA GGGTGAACAC TATCCCATAT
5221 CACCAGCTCA CCGTCTTTCA TTGCCATACG GAATTCCGGA TGAGCATTCA TCAGGCGGGC
5281 AAGAATGTGA ATAAAGGCCG GATAAAACTT GTGCTTATTT TTCTTTACGG TCTTTAAAAA
5341 GGCCGTAATA TCCAGCTGAA CGGTCTGGTT ATAGGTACAT TGAGCAACTG ACTGAAATGC
5401 CTCAAAATGT TCTTTACGAT GCCATTGGGA TATATCAACG GTGGTATATC CAGTGATTTT
5461 TTTCTCCATt ttagcttcct tagctcctga aaatctcgat aactcaaaaa atacgcccgg
5521 tagtgatctt atttcattat ggtgaaagtt ggaacctctt acgtgccgat caacgtctca
5581 ttttcgccaa aagttggccc agggcttccc ggtatcaaca gggacaccag gatttattta
5641 ttctgcgaag tgatcttccg tcacaggtat ttattcggcg caaagtgcgt cgggtgatgc
5701 tgccaactta ctgatttagt gtatgatggt gtttttgagg tgctccagtg gcttctgttt
5761 ctatcagctg tccctcctgt tcagctactg acggggtggt gcgtaacggc aaaagcaccg
5821 ccggacatca gcgctagcgg agtgtatact ggcttactat gttggcactg atgagggtgt
5881 cagtgaagtg cttcatgtgg caggagaaaa aaggctgcac cggtgcgtca gcagaatatg
5941 tgatacagga tatattccgc ttcctcgctc actgactcgc tacgctcggt cgttcgactg
6001 cggcgagcgg aaatggctta cgaacggggc ggagatttcc tggaagatgc caggaagata
6061 cttaacaggg aagtgagagg gccgcggcaa agccgttttt ccataggctc cgcccccctg
6121 acaagcatca cgaaatctga cgctcaaatc agtggtggcg aaacccgaca ggactataaa
6181 gataccaggc gtttccccct ggcggctccc tcgtgcgctc tcctgttcct gcctttcggt
6241 ttaccggtgt cattccgctg ttatggccgc gtttgtctca ttccacgcct gacactcagt
6301 tccgggtagg cagttcgctc caagctggac tgtatgcacg aacccccgt tcagtccgac
6361 cgctgcgcct tatccggtaa ctatcgtctt gagtccaacc cggaaagaca tgcaaaagca

6421 ccactggcag cagccactgg taattgattt agaggagtta gtcttgaagt catgcgccgg
6481 ttaaggctaa actgaaagga caagttttgg tgactgcgct cctccaagcc agttacctcg
6541 gttcaaagag

Comments

ZKRS ORF: 1986-3350 (highlighted in pink)

PCR primer ZKRS-s: 2111-2134 (underlined yellow letters)

PCR primer ZKRS-as: 3174-3194 (complementary strand; underlined yellow letters)

$tRNA^{Pyl}_{CGC}$: 3771-3896 (complementary strand; highlighted in deep green. The anticodon region is highlighted in yellow-green)

$tRNA^{Pyl}_{GGU}$: 4152-4298 (complementary strand; highlighted in deep green. The anticodon region is highlighted in yellow-green)

cat ORF: 4810-5469 (complementary strand; highlighted in light blue)

p15A origin: 5831-192 (highlighted in gray)

Figure S3.

Nucleotide sequence of the substitute plasmid, pACYC184, used in the plasmid exchange experiment shown in Figure 2. The positions and sequences of the PCR primers specific for the substitute plasmids (Tet-s and Tet-as) used in Figure 2e are also shown.

1 GAATTCCGGA TGAGCATTCA TCAGGCGGGC AAGAATGTGA ATAAAGGCCG GATAAAACTT
61 GTGCTTATTT TTCTTTACGG TCTTTAAAAA GGCCGTAATA TCCAGCTGAA CGGTCTGGTT
121 ATAGGTACAT TGAGCAACTG ACTGAAATGC CTCAAAATGT TCTTTACGAT GCCATTGGGA
181 TATATCAACG GTGGTATATC CAGTGATTTT TTTCTCCATt ttagcttcct tagctcctga
241 aaatctcgat aactcaaaaa atacgcccgg tagtgatctt atttcattat ggtgaaagtt
301 ggaacctctt acgtgccgat caacgtctca ttttcgccaa aagttggccc agggcttccc
361 ggtatcaaca gggacaccag gatttattta ttctgcgaag tgatcttccg tcacaggtat
421 ttattcggcg caaagtgcgt cgggtgatgc tgccaactta ctgatttagt gtatgatggt
481 gtttttgagg tgctccagtg gcttctgttt ctatcagctg tccctcctgt tcagctactg
541 acggggtggt gcgtaacggc aaaagcaccg ccggacatca gcgctagcgg agtgtatact
601 ggcttactat gttggcactg atgagggtgt cagtgaagtg cttcatgtgg caggagaaaa
661 aaggctgcac cggtgcgtca gcagaatatg tgatacagga tatattccgc ttcctcgctc
721 actgactcgc tacgctcggt cgttcgactg cggcgagcgg aaatggctta cgaacggggc
781 ggagatttcc tggaagatgc caggaagata cttaacaggg aagtgagagg gccgcggcaa
841 agccgttttt ccataggctc cgcccccctg acaagcatca cgaaatctga cgctcaaatc
901 agtggtggcg aaacccgaca ggactataaa gataccaggc gtttccccct ggcggctccc
961 tcgtgcgctc tcctgttcct gcctttcggt ttaccggtgt cattccgctg ttatggccgc
1021 gtttgtctca ttccacgcct gacactcagt tccgggtagg cagttcgctc caagctggac
1081 tgtatgcacg aaccccccgt tcagtccgac cgctgcgcct tatccggtaa ctatcgtctt
1141 gagtccaacc cggaaagaca tgcaaaagca ccactggcag cagccactgg taattgattt
1201 agaggagtta gtcttgaagt catgcgccgg ttaaggctaa actgaaagga caagttttgg
1261 tgactgcgct cctccaagcc agttacctcg gttcaaagag ttggtagctc agagaacctt
1321 cgaaaaaccg ccctgcaagg cggttttttc gttttcagag caagagatta cgcgcagacc
1381 aaaacgatct caagaagatc atcttattaa tcagataaaa tatttctaga tttcagtgca
1441 atttatctct tcaaatgtag cacctgaagt cagccccata cgatataagt tgtaattctc
1501 atgtttgaca gcttatcatc gataagcttt aatgcggtag tttatcacag ttaaattgct
1561 aacgcagtca ggcaccgtgt ATGAAATCTA ACAATGCGCT CATCGTCATC CTCGGCACCG
1621 TCACCCTGGA TGCTGTAGGC ATAGGCTTGG TTATGCCGGT ACTGCCGGGC CTCTTGCGGG
1681 ATATCGTCCA TTCCGACAGC ATCGCCAGTC ACTATGGCGT GCTGCTAGCG CTATATGCGT
1741 TGATGCAATT TCTATGCGCA CCCGTTCTCG GAGCACTGTC CGACCGCTTT GGCCGCCGCC
1801 CAGTCCTGCT CGGTTCGCTA CTTGGAGCCA CTATCGACTA CGCGATCATG GCGACCACAC
1861 CCGTCCTGTG GATCCTCTAC GCCGGACGCA TCGTGGCCGG CATCACCGGC GCCACAGGTG
1921 CGGTTGCTGG CGCCTATATC GCCGACATCA CCGATGGGGA AGATCGGGCT CGCCACTTCG
1981 GGCTCATGAG CGCTTGTTTC GGCGTGGGTA TGGTGGCAGG CCCCGTGGCC GGGGGACTGT
2041 TGGGCGCCAT CTCCTTGCAT GCACCATTCC TTGCGGCGGC GGTGCTCAAC GGCCTCAACC
2101 TACTACTGGG CTGCTTCCTA ATGCAGGAGT CGCATAAGGG AGAGCGTCGA CCGATGCCCT

2161 TGAGAGCCTT CAACCCAGTC AGCTCCTTCC GGTGGGCGCG GGGCATGACT ATCGTCGCCG
2221 CACTTATGAC TGTCTTCTTT ATCATGCAAC TCGTAGGACA GGTGCCGGCA GCGCTCTGGG
2281 TCATTTTCGG CGAGGACCGC TTTCGCTGGA GCGCGACGAT GATCGGCCTG TCGCTTGCGG
2341 TATTCGGAAT CTTGCACGCC CTCGCTCAAG CCTTCGTCAC TGGTCCCGCC ACCAAACGTT
2401 TCGGCGAGAA GCAGGCCATT ATCGCCGGCA TGGCGGCCGA CGCGCTGGGC TACGTCTTGC
2461 TGGCGTTCGC GACGCGAGGC TGGATGGCCT TCCCCATTAT GATTCTTCTC GCTTCCGGCG
2521 GCATCGGGAT GCCCGCGTTG CAGGCCATGC TGTCCAGGCA GGTAGATGAC GACCATCAGG
2581 GACAGCTTCA AGGATCGCTC GCGGCTCTTA CCAGCCTAAC TTCGATCACT GGACCGCTGA
2641 TCGTCACGGC GATTTATGCC GCCTCGGCGA GCACATGGAA CGGGTTGGCA TGGATTGTAG
2701 GCGCCGCCCT ATACCTTGTC TGCCTCCCCG CGTTGCGTCG CGGTGCATGG AGCCGGGCCA
2761 CCTCGACCTG Aatggaagcc ggcggcacct cgctaacgga ttcaccactc caagaattgg
2821 agccaatcaa ttcttgcgga gaactgtgaa tgcgcaaacc aacccttggc agaacatatc
2881 catcgcgtcc gccatctcca gcagccgcac gcggcgcatc tcgggcagcg ttgggtcctg
2941 gccacgggtg cgcatgatcg tgctcctgtc gttgaggacc cggctaggct ggcggggttg
3001 ccttactggt tagcagaatg aatcaccgat acgcgagcga acgtgaagcg actgctgctg
3061 caaaacgtct gcgacctgag caacaacatg aatggtcttc ggtttccgtg tttcgtaaag
3121 tctggaaacg cggaagtccc ctacgtgctg ctgaagttgc ccgcaacaga gagtggaacc
3181 aaccggtgat accacgatac tatgactgag agtcaacgcc atgagcggcc tcatttctta
3241 ttctgagtta caacagtccg caccgctgtc cggtagctcc ttccggtggg cgcggggcat
3301 gactatcgtc gccgcactta tgactgtctt ctttatcatg caactcgtag gacaggtgcc
3361 ggcagcgccc aacagtcccc cggccacggg gcctgccacc atacccacgc cgaaacaagc
3421 gccctgcacc attatgttcc ggatctgcat cgcaggatgc tgctggctac cctgtggaac
3481 acctacatct gtattaacga agcgctaacc gtttttatca ggctctggga ggcagaataa
3541 atgatcatat cgtcaattat tacctccacg gggagagcct gagcaaactg gcctcaggca
3601 tttgagaagc acacggtcac actgcttccg gtagtcaata aaccggtaaa ccagcaatag
3661 acataagcgg ctatttaacg accctgccct gaaccgacga ccgggtcgaa tttgctttcg
3721 aatttctgcc attcatccgc ttattatcac ttattcaggc gtagcaccag gcgtttaagg
3781 gcaccaataa ctgccttaaa aaaaTTACGC CCCGCCCTGC CACTCATCGC AGTACTGTTG
3841 TAATTCATTA AGCATTCTGC CGACATGGAA GCCATCACAG ACGGCATGAT GAACCTGAAT
3901 CGCCAGCGGC ATCAGCACCT TGTCGCCTTG CGTATAATAT TTGCCCATGG TGAAAACGGG
3961 GGCGAAGAAG TTGTCCATAT TGGCCACGTT TAAATCAAAA CTGGTGAAAC TCACCCAGGG
4021 ATTGGCTGAG ACGAAAAACA TATTCTCAAT AAACCCTTTA GGGAAATAGG CCAGGTTTTC
4081 ACCGTAACAC GCCACATCTT GCGAATATAT GTGTAGAAAC TGCCGGAAAT CGTCGTGGTA
4141 TTCACTCCAG AGCGATGAAA ACGTTTCAGT TTGCTCATGG AAAACGGTGT AACAAGGGTG
4201 AACACTATCC CATATCACCA GCTCACCGTC TTTCATTGCC ATACG

Comments

p15A origin: 581-1493 (highlighted in gray)

Tetracycline resistance gene ORF: 1581-2771 (highlighted in yellow-green)

PCR primer Tet-s: 1805-1823 (underlined red letters)

PCR primer Tet-as: 2241-2262 (complementary strand; underlined red letters)

cat ORF: 3805-219 (complementary strand; highlighted in light blue)
